# Comparative Analysis of Rice Yield and Economic Performance Across Different Planting Patterns in Double-Cropping Rice Systems Under Global Warming

**DOI:** 10.3390/plants14233593

**Published:** 2025-11-25

**Authors:** Qianxing Su, Jinyao Wang, Weisheng Lv, Ming Chen, Wen Xiong, Le Chen, Yongjun Zeng

**Affiliations:** 1 Ministry of Education and Jiangxi Key Laboratory of Crop Physiology, Ecology and Genetic Breeding, Jiangxi Agricultural University, Nanchang 330045, China; 2 College of Ceramic Art and Design, Jingdezhen University, Jingdezhen 333400, China; 3 Jiangxi Red Soil and Germplasm Resources Institute, Nanchang 331717, China

**Keywords:** global warming, double-cropping rice area, planting pattern, yield, economic benefit

## Abstract

Under global warming, the differences in yield, soil nutrients, and economic benefits between various planting patterns in double-cropping rice areas were compared, and the high-yield and high-efficiency planting patterns that can adapt to climate change were selected. Four planting patterns, namely rape–rice (RaR), fallow–rice (FR), Chinese milk vetch–early rice–late rice (CRR), and fallow–early rice–late rice (FRR), were investigated. Compared with FRR, the yield of early rice increased by 13.6% using the CRR rotation. CRR could increase the spikelet per panicle of early rice, thereby enhancing rice yield. The soil’s available nitrogen content demonstrated an increase under the CRR rotation when compared with FRR. The yield under RaR increased by 11.9% on average compared with FR. The RaR rotation enhanced panicles per ha, thereby increasing rice yield. RaR could increase the soil’s available nutrient content compared with FR. Compared with CRR, FRR, and FR, the net income of RaR was higher by 1031 CNY/ha, 2046 CNY/ha and 5762 CNY/ha, respectively. Comprehensively compared with the other three planting patterns, RaR could effectively improve the soil fertility of paddy fields, grain yield, and net income. RaR is a sustainable planting pattern with a high yield and high efficiency worth popularizing. In addition, under the warming climate, the growth period and sowing date of rice of RaR and FR should be appropriately extended and postponed to avoid encountering more frequent high-temperature weather.

## 1. Introduction

Rice (Oryza sativa) is a staple food in China, and rice accounts for 33.8% of the Chinese grain area [1]. The double-cropping of rice is an important agricultural planting pattern that fully utilizes local temperature and light resources and improves land utilization rate and grain output in southern China. The cultivation of double-cropped rice has provided an important contribution to increasing grain production and ensuring food security [2]. However, against the background of global climate warming [3], the risks faced by agricultural production are increasing continuously. Global warming significantly impacts crop production and poses a serious threat to food security due to increasing temperatures, irregular rainfall regimes, extreme weather events, insufficient winter chilling, and water scarcity [4,5,6]. Moreover, climate change is a possible factor impacting the geospatial and temporal changes in the actual rice-cropping area in China [7]. As an important region for ensuring food security in China, the adaptable adjustment of planting patterns in double-cropping rice areas is crucial for stabilizing grain yield and ensuring food security.

Global warming has significantly changed the climatic conditions within the double-cropped rice growing season by, for example, raising the average temperature during the growth period, accelerating the growth process of rice, and shortening the reproductive period [8], which has an effect on the potential productivity, yield, and quality of rice [9,10,11]. Zhang et al. [12] found that global warming significantly reduced the yield of early rice and late rice. Meanwhile, the safe production dates of double-cropped rice also change due to climate change. Lü et al. [13] found that the daily mean temperature increased significantly, accumulated temperature increased remarkably, and sunshine hours changed slightly during the safe production phases. Ai et al. [14] also proposed that the obvious advance of the safe sowing dates and the safe transplanting dates of early rice in the watered nursery facilitates early sowing, increasing yield potential except the rest of safe production dates.

Global warming has also led to the expansion of potential double-cropped areas [15], which is beneficial for increasing the annual crop yield per unit area. However, the single-cropped system fails to fully utilize the temperature and light resources throughout the year, thus reducing the land utilization rate. Compared with the single-cropped rice system, the double-cropped rice system increases the yield of an additional rice-cropping season and the utilization of temperature and light resources, but it may give rise to soil acidification, soil degradation, and a decline in soil fertility. Both of the above two models are in winter fallow, which not only wastes valuable light and heat resources but also accelerates soil erosion due to soil exposure, resulting in the depletion of organic matter and essential nutrients [16]. However, the utilization of winter cover crops not only enhances rice yields but also plays a role in soil carbon sequestration, thereby supporting sustainable rice farming and mitigating soil degradation. Chinese milk vetch is a widely cultivated leguminous green manure in southern China. It makes a significant contribution to improving soil fertility and productivity [17,18], enhancing soil health, replacing chemical fertilizers [19]. The previous research also indicated that for double-rice cropping areas, it is recommended to use Chinese milk vetch with 80% chemical fertilizer to enhance rice yield, crop nutrient uptake, and the utilization efficiency of N, P, and K fertilizers [20]. The rice–rape rotation is a high-yield, high-efficiency, environmentally friendly agricultural rotation planting system [21]. These possess advantages such as high annual yield per unit land area, high land use efficiency, high light and temperature utilization rates, and significant economic benefits. Moreover, they are of great significance for ensuring the secure production of food and edible oil in China within limited land resources. But the spatial variability of climate may bring uncertainties in terms of scaling up site-specific field management strategies to the regional level. The irrigation requirements [22], planting dates [23], crop varieties [24], insect pests [25,26], etc., which considerably influence crop yield, should be considered, during the selection of planting patterns. Meanwhile, Yang et al. [27] also Indicated that the average daily temperature and effective accumulated temperature were the most significant climate factors affecting yield, and proposed that mechanical direct seeding under vegetable–rice rotation pattern and mechanical transplanting under rape–rice or wheat–rice were the rice planting methods that optimized the climate resources. To address the impacts brought by global climate warming, Mohapatra et al. [28] conducted a climate smartness assessment of different cropping patterns in most areas of planting rice under global warming, and proposed that incorporating legumes and oil seed crops such as sunflower would substantially increase the climate smartness compared to rice mono-cropping. Moreover, in China, previous research mainly concentrated on the carbon footprint and greenhouse gas emissions of various planting patterns [29,30]. There have been limited studies on the climate adaptability, yield, and economic benefits of different cropping patterns under global warming. Thus, we put forward the hypothesis that the rape–rice or rice–rice–green manure cropping patterns may be more suitable for the current environment.

This research discovered that, in comparison with 2021, the extreme high temperatures in 2022 had a severe impact on rice production. Regarding issues such as extremely high temperatures resulting from global warming and the decline in the annual productivity of paddy fields, the differences in yield, soil nutrient and economic benefits among various planting patterns in double-cropping rice area were compared, and the high-yield and high-efficiency planting patterns that can adapt to climate change were selected, which was of great significance for ensuring stable grain yield in the double-cropping rice area and promoting sustainable agricultural development.

## 2. Results

### 2.1. The Effect of Different Planting Patterns on Rice Yield and Annual Grain Yield

#### 2.1.1. Yield and Its Composition

Compared with FRR, the yield of early rice of CRR increased by 13.6% in 2022 (Table 1), while it was not significant in 2021. Compared to FRR, CRR had no significant impact on the composition of early rice yield in 2021. However, in 2022, the number of grains per panicle increased by 11.58%. Regarding late rice, CRR had no significant effect on the yield and yield components compared to FRR. In contrast, compared to FR, RaR led to an increase in rice yield by 13.5% and 10.3% in 2021 and 2022, respectively. The total panicle number per hectare increased, yet the effect was not significant. Unlike in 2021, the heading period of both mid-season rice and late rice was affected by extreme high temperatures , resulting in a significant decline in the grain setting rate.

#### 2.1.2. Annual Grain Yield

**Figure 1:**
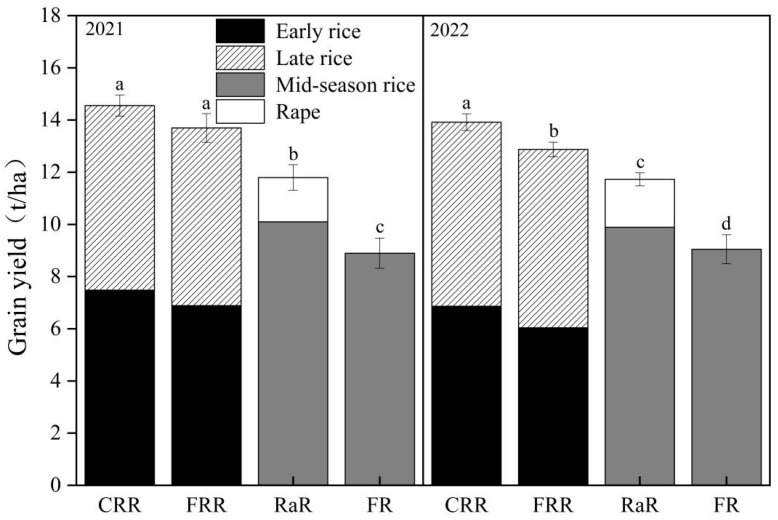
Comparison of annual grain yield of different planting patterns. Note: Different lowercase letters indicate a significant difference between different treatments in the same year and the same season (*p* ≤ 0.05). Rape–rice (RaR), fallow–rice (FR), Chinese milk vetch–early rice–late rice (CRR), and fallow–early rice–late rice (FRR).

In terms of the annual grain yield for past two years, CRR > FRR > RaR > FR (Figure 1). In 2021, the total grain yield of CRR reached 14.56 t/ha, which was 23.39% higher than that of RaR and 63.60% higher than that of FR, respectively. Compared with FR, RaR led to a 29.61% increase in grain yield. In 2022, the total yield of CRR was 13.92 t/ha, which was 8.16%, 18.67%, and 54.03% higher than that of FRR, RaR, and FR, respectively. Compared with FR, RaR also increased the grain yield by 29.61%.

#### 2.1.3. Annual Biomass

**Figure 2:**
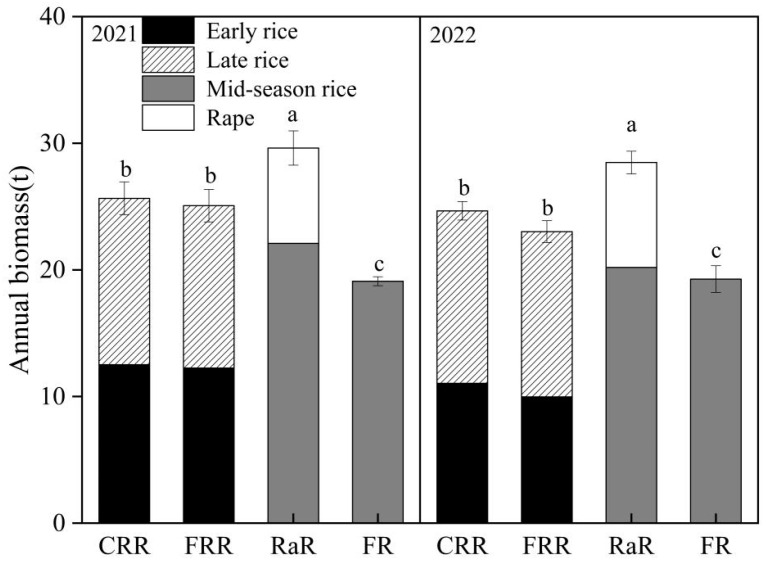
Comparison of annual biomass of different planting patterns. Note: Different lowercase letters indicate a significant difference between different treatments in the same year and the same season (*p* ≤ 0.05). Rape–rice (RaR), fallow–rice (FR), Chinese milk vetch–early rice–late rice (CRR), and fallow–early rice–late rice (FRR).

In 2021 and 2022 (Figure 2), the annual biomass of RaR was 16.80% and 55.00%, 19.46% and 47.72% higher than that of the double-cropped rice pattern and FR, respectively. In 2021 and 2022, the annual biomass of the double-cropped rice pattern was 32.71% and 23.65% higher than that of FR, respectively. There was no significant difference between CRR and FRR in the two years.

### 2.2. The Available Nutrient of Soil

**Figure 3:**
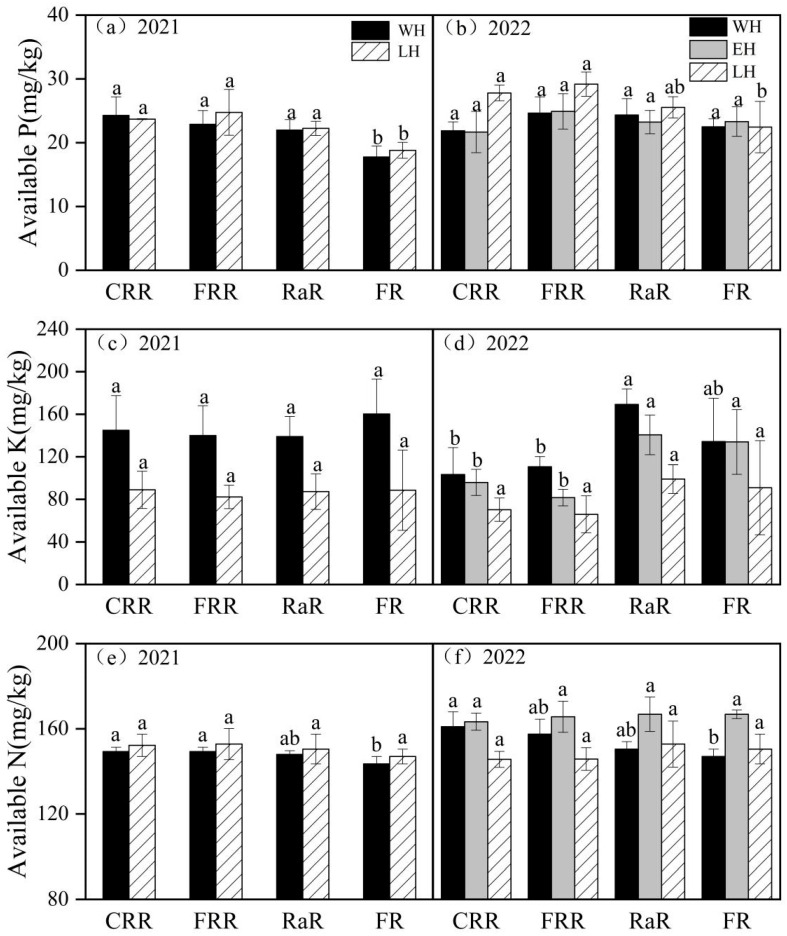
Effects of different planting patterns on available N, P and K. Note: Different lowercase letters indicate a significant difference between different treatments in the same year and the same season (*p* ≤ 0.05). Rape–rice (RaR), fallow–rice (FR), Chinese milk vetch–early rice–late rice (CRR), and fallow–early rice–late rice (FRR). WH: the Chinese milk vetch blooming period and after the harvest of rape; EH: after the harvest of early rice; LH: after the harvest of late rice and rice. (**a**) and (**b**) represent the available P content in 2021 and 2022. (**c**) and (**d**) represent the available K content in 2021 and 2022. (**e**) and (**f**) represent the available N content in 2021 and 2022.

Compared with FRR (Figure 3), the available P of CRR in the soil was not increased significantly in the past two years. In 2021, the available P of RaR in the soil was increased significantly in WH and LH, compared with FR. In 2022, compared with FR, available P of RaR in the soil was increased but had no significant effect.

The content of available K in the soil showed a decreasing trend, WH > EH > LH. Compared with FRR, the available K of CRR in the soil was not increased significantly in the past two years. In 2021, there was no significant difference in the available K of soil between RaR and FR. In 2022, compared with FR, available K of RaR in the soil was increased but had no significant effect.

In 2021, there was no significant difference in the available N of soil between CRR and FRR. In 2022, compared with FRR, available N of CRR in the soil was increased in WH but had no significant effect. Compared with FR, the available N of RaR in the soil was increased in WH but had no significant effect.

### 2.3. The Effect of Different Planting Patterns on Economic Benefits

In terms of the total cost of crops, artificial transplanting mid-season rice > direct-seeding mid-season rice > late rice > early rice > rape > Chinese milk vetch (Table 2). Among different planting patterns, the total annual cost in 2021 was CRR > FRR > RaR > FR. Compared with CRR and FRR, the total cost of RaR was decreased by CNY 2911/ha and CNY 1638/ha, respectively. In 2022, the total annual cost was CRR > RaR > FRR > IR. In the past two years, the cost of CRR was the highest, among which the labor cost was the highest, at CNY 11,169/ha.

In terms of output value, mid-season rice > late rice > early rice > rape. In terms of total output value, CRR (37,697 CNY/ha) > RaR (35,778 CNY/ha) > FRR (35,588 CNY/ha) > FR (22,250 CNY/ha) in 2021. The total output value of CRR was CNY 1919/ha, CNY 2109/ha and CNY 15,447/ha higher than FRR, RaR and FR, respectively. In 2022, the total output value was RaR (CNY 36,334/ha) > CRR (CNY 36,255/ha) > FRR (CNY 33,788/ha) > FR (CNY 22,625/ha). Compared with CRR, FRR and FR, the total output value of RaR was increased by CNY 79/ha, CNY 2546/ha and CNY 13,849/ha. In terms of net income for past two years: RaR > CRR > FRR > FR. In 2021 and 2022, compared with CRR, FRR and FR, the net income of RaR was increased by CNY 992/ha, CNY 1828/ha and CNY 5672/ha, CNY 1070/ha, CNY 2264/ha and CNY 5852/ha, respectively. The net income of RaR was highest.

## 3. Discussion

### 3.1. The Analysis of Different Planting Patterns on Grain Yield and Yield Formation

Crop biomass serves as the material foundation for yield formation. Within a specific range, the greater the dry matter accumulation, the higher the grain yield will be [31]. The study revealed that, in the double-cropped rice pattern, mid-season rice exhibited a higher biomass accumulation and grain yield compared to early and late rice. Nevertheless, the increase in the dry matter accumulation and dry matter production capacity of crops was primarily attributed to the extension of the growth period. Compared with a decade ago, this study selected rice varieties with a longer growth period in RaR, thus enhancing the rice yield and the annual grain yield [32]. Moreover, in comparison with the single-cropping of FR, the yield of rape was directly enhanced, and the yield and dry matter accumulation of rice were indirectly increased in RaR rotation, respectively (Table 1, Figure 1). Planting Chinese milk vetch in winter fallow farmlands also indirectly increased the yield of early rice in CRR. Compared with traditional planting methods, planting rape or leguminous green manure in winter fallow farmlands can increase the rice yield and the annual grain yield. Meanwhile, several studies have shown that the average winter temperature in China has risen over the past 60 years (1961–2020), and the diurnal temperature range has decreased [33,34], which was conducive to the growth of winter crops.

Our research indicated that, across two cropping cycles, the grain yields subsequent to growing rape and Chinese milk vetch were higher than those of rice grown after fallow. This finding was in line with previous research indicating that rape is a beneficial rotation crop for sustaining the high productivity of single-season rice [35], and growing Chinese milk vetch during the fallow season is a viable approach to maintain rice productivity in both double-cropping systems [36,37,38]. More significantly, our study indicated that planting rape can more effectively enhance productivity. The higher grain yield of rice grown after rape and Chinese milk vetch was due to improvements in both sink size and source capacity. For the sink size, planting rape and Chinese milk vetch increased panicle number of rice, and number of spikelet per panicle of early rice, respectively (Table 1). However, another study determined that growing rape increased panicle size (spikelet number per panicle) and panicle number in early rice and late rice, respectively, and growing Chinese milk vetch increased panicle number in both the early rice and late rice [39]. These findings indicated that different rice varieties and management methods had different responses to plant rape and Chinese milk vetch in winter.

**Figure 4:**
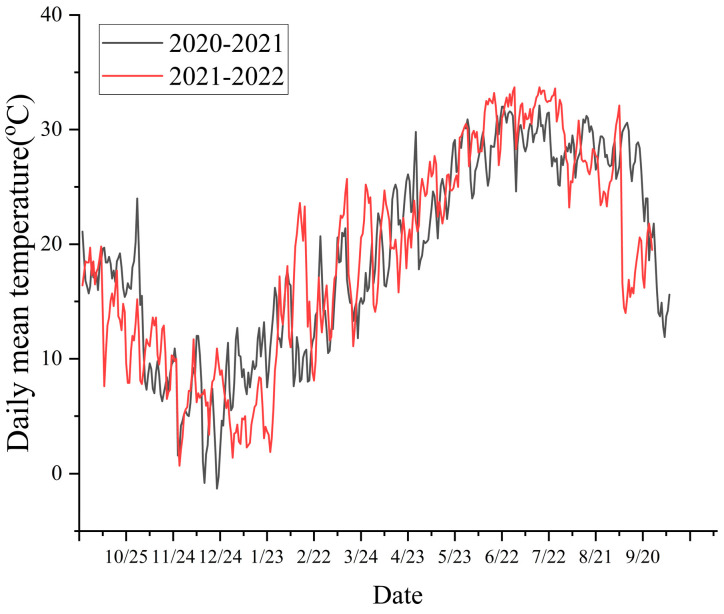
Average daily temperature and rainfall in the test site (October 2020–October 2022).

In 2022, the planting method of RaR was altered from hole direct seeding to artificial transplanting, and the input of nitrogen fertilizer was increased. Nevertheless, the rice failed to achieve the expected yield. This was because the seed setting rate of the rice decreased significantly, mainly due to the extreme high temperature and drought that occurred during the flowering period (Figure 4). With global warming, extremely high temperatures would be frequent [40,41]. The seed setting rate of rice was decreased due to extreme high temperatures during the heading and flowering period, which was an important reason for the reduction in rice yield [42,43]. Therefore, the heading and flowering periods of rice should be adjusted properly. Selecting mid-season rice varieties with longer growth periods or postponing the sowing date of mid-season rice can help avoid exposure to elevated-temperature weather, thereby reducing the damage caused by extreme temperatures.

### 3.2. The Analysis of Different Planting Patterns on Soil Quality

Previous research also indicated that a close association existed between the rice yield and the nutrient level of the soil [44]. However, cultivating a single type of rice over the long term will deplete soil fertility. The study also indicated that maize–upland rice cultivation with burned maize residues can decrease soil organic carbon [45]. The degradation of soil fertility is not favorable for boosting crop yields and ensuring the sustainability of the land. Winter planting can improve soil fertility and increase the yield of rice [46]. Chinese milk vetch is a promising winter cover crop. It can reduce the reliance on fertilizers and boost crop productivity in southern China. During its growth as organic green manure, Chinese milk vetch can fix atmospheric N2 through the root–rhizobia symbiotic nitrogen fixation system. Once returned to the field, it releases a substantial amount of nitrogen accumulated in its tissues for rice to absorb and utilize. The abundant supply of nitrogen can promote the absorption and utilization of phosphorus and potassium by rice, thereby increasing rice yield [47]. The study also revealed that the available nitrogen content of CRR in the soil was higher than that of FRR, and it significantly enhanced the yield of early rice. Including legume cover crop in rice-based rotation system improved plant growth and development by altering soil nitrogen forms plus ameliorating soil microbial communities and antioxidant system, which alleviates oxidative damage in plants [37]. Furthermore, the combination of Chinese milk vetch and straw incorporation into the soil exerted a positive influence on soil bacterial diversity and structure, particularly on beneficial microorganisms. This contributed to the enhancement of soil biological fertility [48]. Similar to Chinese milk vetch, planting rape also improved soil fertility and increased rice yield. However, their effects on the soil differed. Previous research indicated that using rape as a preceding crop in a rotation system could significantly boost rice yield and the soil’s indigenous nutrient supply capacity [35]. The rape–rice rotation and rape–green manure–rice rotation of all increased phosphorus nutrients of red soil and promoted phosphorus availability [49,50]. The rice–rape rotations contributed to the improvement of soil quality. This was achieved by increasing the organic matter, total nitrogen content, and the activities of soil sucrase, phosphatase, urease, dehydrogenase, and cellulase. Additionally, these rotations inhibited the decline in pH, available nitrogen, and phosphorous contents [51]. Meanwhile, the recent studies indicated that the rape–rice rotation system improved soil structural stability and sustainability of soil fertility by increased soil organic carbon and sequestration of soil organic nitrogen [52,53]. The aforementioned studies indicated that cultivating Chinese milk vetch and rape in winter was a crucial rotation system for enhancing the sustainability of soil fertility.

### 3.3. Economic Benefit of Different Planting Patterns

Compared with FRR, although CRR led to an increase in the cost of Chinese milk vetch (1273 CNY/ha), the output value of double-cropping rice and the net income were higher than those of FRR. In the past two years, the annual net income of CRR was CNY 836/ha and CNY 1194/ha higher than that of FRR, respectively. The study suggested that the increase in net income in CRR was attributed to the increased yield of early rice. However, the increase in net income in RaR was achieved by increasing the yields of both rice and rape. Our research indicated that winter planting could enhance economic benefits. The previous study also proved that compared with the single cropping system, the double cropping system had significant advantages in annual rice yield, dry matter accumulation, total economic benefit, light and effective accumulated temperature distribution rate [54].

In 2021 and 2022, when compared with CRR, FRR, and FR, the net income of RR increased by CNY 992/ha, CNY 1828/ha, and CNY 5672/ha in 2021, and by CNY 1070/ha, CNY 2264/ha, and CNY 5852/ha in 2022, respectively. The net income of RaR was the highest. Even though the rice yield decreased under the condition of extreme high temperature in 2022, RaR still attained a relatively high income (CNY 10,444/ha). A previous study concluded that, for the purpose of maximizing economic profit while maintaining sustainable agriculture in areas of the middle and lower reaches of the Yangtze River that are sensitive to climate variability, the single rice system is superior to the double rice system [55]. However, our results showed that compared with FR, RaR could be superior to the double rice systems for the sake of maximizing economic profit under global warming. Zhao et al. [56] indicated that the economic income of the rape–rice pattern reached CNY 14,468/ha. This was because his experiment adopted the method of no-till broadcasting for both rape and rice, which greatly reduced labor costs and thus improved economic benefits. The recent study also suggested that the net economic return of direct seeded rice–direct seeded rape rotation system was higher than traditional transplanting rice–direct seeded rape rotation system [21]. The aforementioned studies demonstrated that the rape–rice system was a planting pattern with significant economic benefits in southern China. However, the crucial factor for increasing the economic income of the RaR system lies in optimizing the planting method without compromising crop yields and reducing labor costs.

## 4. Materials and Methods

### 4.1. Experimental Site Description

The experiment began in 2020 at the Jiangxi Red Soil and Germplasm Resources Institute (Zhanggong Town, Jinxian County, Nanchang City, Jiangxi Province, 116°10′6″ E, 28°21′31″ N). The experimental site is located in a subtropical monsoon climate zone. Compared to 2021, the rice in 2022 was suffering from high temperatures during the heading and flowering period (Figure 1). Soil samples were collected from the 0 to 20 cm soil depth. The physico-chemical properties of the soil were pH 5.27, organic matter 27.31 g/kg, total N 1.88 g/kg, available P 25.4 mg/kg, available K 119 mg/kg, and available N 150.5 mg/kg, respectively.

### 4.2. Experimental Materials and Design

The crop varieties were consistent for the past two years. The early rice variety was Xiangzaoxian 45, the late rice variety was Meixiangzhan 2, the mid-season rice variety was Jiafengyou 2, the rape variety was Fengyou 730, and the Chinese milk vetch was Yujiang Daye. Four planting patterns were selected: rape–rice (RaR), fallow–rice (FR), Chinese milk vetch–early rice–late rice (CRR), and fallow–early rice–late rice (FRR). The experiment was laid out as a randomized complete block design. Each treatment had three replicates and each plot was 90 m^2^ (15 m × 6 m). The crops were harvested, with full straw returning to the field. Chinese milk vetch seeds were manually broadcast, and then plowed back into the field during the Chinese milk vetch blooming period. The field management of different crops is shown in Table 3.

### 4.3. Sampling and Analysis

#### 4.3.1. Crop Yield

At the maturity stage of rice, 200 hills from the center of each subplot were harvested for the grain yield determination. Five hills with the mean panicle number from the center of each subplot were sampled to record the different yield components. The panicle number per ha was determined by counting the panicle number per hill and multiplying it by the plant densities. The panicles were hand-threshed, air-dried, and then the structure of the panicle was examined using the water-float method [57]. At the maturity stage of rape, five hills from the center of each subplot were sampled to record the different yield components. The number of hills within 1 m^2^ was randomly measured three times to calculate the harvest density. The rape in the plot was both harvested and dried, then the actual yield was calculated.

#### 4.3.2. Dry Matter Production

At the maturity stage of rice, five hills with the mean panicle number from the center of each subplot were sampled to record the biomass. The roots were cut off from the base, and the stems, leaves, and panicles were separated, wrapped in paper, and placed in a drying oven at 105 °C for half an hour, and then dried at 70 °C until constant weight, and weighed.

#### 4.3.3. The Available Nutrient of Soil

Soil samples were collected from the 0–20 cm soil layer during the Chinese milk vetch blooming period, and after the harvest of rape, early rice, late rice and mid-season rice. Five soil samples from each plot were pooled to create a composite sample. After air-drying and sieving (0.25 mm), soil samples were stored in a Ziplock bag. The contents of available nitrogen, phosphorus and potassium in the soil samples were determined by the measurement methods described in Soil and Agricultural Chemical Analysis [58].

#### 4.3.4. Agricultural Input Costs

The total cost included the costs of seeds, pesticides, fertilizers, labor, and machinery (Table 4). The man-hours were in raising rice seedlings and field management. Grain subsidies are not included. Net income = output value − total cost. Output value = crop yield × crop price. Grain subsidies were not included. Net income = output value − total cost. Output value = crop yield × crop price.

### 4.4. Statistics and Analysis

The experimental data were statistically analyzed by Microsoft Excel 2019 and SPSS 25.0 and variance tests were performed using the LSD method at *p* < 0.05. Graphs were produced using Origin 2018 software.

## 5. Conclusions

The pattern of CRR improved the content of available N and spikelet per panicle, which in turn increased the early rice yield, annual grain yield, and economic benefit. The pattern of RaR improved the content of available nutrient and panicles per ha, thereby increasing rice yield, and improved the annual grain yield, economic benefit with an increase in rape grain yield in fallow. Comprehensively compared with the other three planting patterns, RaR can effectively improve the soil fertility of the paddy field, annual grain yield and net income. RaR was a sustainable planting pattern with high yield and high efficiency worthy popularizing. Under the background of climate warming, the growth period and sowing date of rice of RaR and IR should be appropriately extended and postponed to avoid encountering more frequent high-temperature weather.

## Figures and Tables

**Table 1:** Effects of different planting patterns on rice yield and its composition.

Year	Season	Treatment	Panicles (10^4^/ha)	Spikelets per Panicle	Seed Setting Rate (%)	1000-Grain Weight (g)	Yield (t/ha)
2021	Early rice	CRR	396 a	101 a	83.23 a	24.80 a	7.48 a
		FRR	404 a	94 a	81.89 a	24.44 a	6.89 a
	Late rice	CRR	284 a	151 a	92.44 a	20.59 a	7.08 a
		FRR	286 a	148 a	88.75 a	20.33 a	6.81 a
	Mid-season rice	RaR	190 a	286 a	83.79 a	26.16 a	10.10 a
		FR	171 a	278 a	84.17 a	25.94 a	8.90 b
2022	Early rice	CRR	299 a	106 a	89.97 a	25.40 a	6.86 a
		FRR	301 a	95 b	89.62 a	26.27 a	6.04 b
	Late rice	CRR	364 a	138 a	73.10 a	20.39 a	7.05 a
		FRR	358 a	137 a	70.77 a	20.58 a	6.83 a
	Mid-season rice	RaR	263 a	233 a	70.98 a	26.05 a	9.98 a
		FR	251 a	238 a	67.51 a	26.16 a	9.05 b

Note: Different lowercase letters indicate a significant difference between different treatments in the same year and the same season (*p* ≤ 0.05). Rape–rice (RaR), fallow–rice (FR), Chinese milk vetch–early rice–late rice (CRR), and fallow–early rice–late rice (FRR).

**Table 2:** Effects of different planting patterns on economic benefits (Chinese Yuan (CNY)/ha).

Year	Treatment		Cost (Chinese Yuan)						Total Income (Chinese Yuan)	Net Income (Chinese Yuan)
			Seed	Fertilizer	Pesticide	Labor	Machinery	Total Cost		1st Season + 2nd Season Total
2021	Crops	Early rice	450	2286	1289	4648	2699	11,372		
		Late rice	900	2482	1859	6297	2699	14,236		
		Chinese milk vetch	1048	0	0	225	0	1273		
		rape	360	1919	2279	1349	1949	7856		
		rice (hole direct seeding)	4048	3340	2429	3598	2699	16,114		
2022		rice (transplanting)	3373	3536	2429	5997	2699	18,033		
2021	Planting patterns	CRR	2397	4768	3148	10,945	5397	26,881	37,697	10,816
		FRR	1350	4768	3148	11,169	5397	25,608	35,688	9980
		RaR	4408	5259	4708	4948	4648	23,970	35,778	11,808
		FR	4048	3340	2429	3598	2699	16,114	22,250	6136
2022		CRR	2397	4768	3148	10,945	5397	26,881	36,255	9374
		FRR	1350	4768	3148	11,169	5397	25,608	33,788	8180
		RaR	3733	5455	4708	7346	4648	25,890	36,334	10,444
		FR	3373	3536	2429	5997	2699	18,033	22,625	4592

Note: Rape–rice (RaR), fallow–rice (FR), Chinese milk vetch–early rice–late rice (CRR), and fallow–early rice–late rice (FRR).

**Table 3:** Field management of different crops.

Crop	Sowing Date–Harvest Date	Transplanting Modes and Density	Fertilizer Amount	Fertilizer Methods
Early rice	24 March 2021–14 July 2021	Artificial transplanting 25 cm × 14 cm	N: 120 kg/hm^2^ P_2_O_5_: 75 kg/hm^2^ K_2_O: 120 kg/hm^2^	Nitrogen fertilizer was applied at the rate of basal fertilizer: tillering fertilizer: panicle fertilizer = 5:2:3, phosphorus fertilizer was both applied as basal fertilizer, and potassium fertilizer was applied at the rate of basal fertilizer: panicle fertilizer = 7:3
	28 March 2022–17 July 2022			
Late rice	26 June 2021–14 October 2021	Artificial transplanting 25 cm × 16 cm	N: 150 kg/hm^2^ P_2_O_5_: 75 kg/hm^2^ K_2_O: 120 kg/hm^2^	
	1 July 2022–25 October 2022			
Rape	19 October 2020–29 April 2021	Broadcast sowing 6 kg/hm^2^	N: 120 kg/hm^2^ P_2_O_5_: 33.6 kg/hm^2^ K_2_O: 38.4 kg/hm^2^	The special compound fertilizer was applied to rape as basal fertilizer
	13 October 2021–25 April 2022			
Mid-season rice	8 May 2021–29 September 2021	Hole direct seeding 27 cm × 16 cm	N: 195 kg/hm^2^ P_2_O_5_: 90 kg/hm^2^ K_2_O: 180 kg/hm^2^	Nitrogen fertilizer was applied at the rate of basal fertilizer: tillering fertilizer: panicle fertilizer = 4:2:4, phosphorus fertilizer was both applied as basal fertilizer, and potassium fertilizer was applied at the rate of basal fertilizer: panicle fertilizer = 6:4
	10 May 2022–30 September 2022	Artificial transplanting 30 cm × 16 cm	N: 225 kg/hm^2^ P_2_O_5_: 90 kg/hm^2^ K_2_O: 180 kg/hm^2^	

**Table 4:** Price list of different agricultural inputs.

Crop		Seed Prices (CNY/kg)	Frequency of Medication		Man-Hour (h)	Selling Price (According to Local Market) (CNY/kg)
			Herbicide	Pesticide		
early rice		10	1	2	120	2.2
later rice		20	1	2	120	3
mid-season rice	Direct broadcast	90	1	3	90	2.5
	transplanting				150	
rape		60	1	4	90	6.2
Chinese milk vetch		30			15	
Other cost item		Labor cost (CNY/h)	rape special fertilizer (CNY/kg)	Urea (CNY/kg)	potassium chloride (CNY/kg)	Calcium magnesium phosphate fertilizer (CNY/kg)
		15	4	3	4.4	1
		Tillage of rice (CNY/ha)	Rape ditching (CNY/ha)	Herbicide (CNY/ha)	Pesticides (CNY/ha)	Spraying pesticides by drone (CNY/ha)
		1500	750	150	450	120
		Transplanting (CNY/ha)			Hole direct seeding (CNY/ha)	Harvest of rice and rape (CNY/ha)
		Early rice	Late rice	Mid- season rice	Mid- season rice	
		2850	4500	3750	2250	1500

## Data Availability

The original contributions presented in this study are included in the article. Further inquiries can be directed to the corresponding author.

## Abbreviations

RaR	rape–rice
FR	fallow–rice
CRR	Chinese milk vetch–early rice–late rice
FRR	fallow–early rice–late rice
CNY	Chinese Yuan
